# Data on concentrations of polycyclic aromatic hydrocarbons (PAHs) in roasted and fried chicken – A case study: Bushehr, Iran

**DOI:** 10.1016/j.dib.2018.11.012

**Published:** 2018-11-06

**Authors:** Hossein Arfaeinia, Elhameh Cheshmazar, Kamaladdin Karimyan, Mohammad Darvishmotevalli, Seyed Enayat Hashemi

**Affiliations:** a Department of Environmental Health Engineering, School of Public Health, Bushehr University of Medical Sciences, Bushehr, Iran; b Department of Nutrition, School of Public Health, Iran University of Medical Sciences, Tehran, Iran; c Environmental Health Research Center, Kurdistan University of Medical Sciences, Sanandaj, Iran; d Department of Environmental Health Engineering, Public Health School, Isfahan University of Medical Sciences, Isfahan, Iran

**Keywords:** Polycyclic aromatic hydrocarbon (PAH), Roasted chicken, Fried chicken, Bushehr

## Abstract

In the present data article, the presence and amount of 16 polycyclic aromatic hydrocarbons (PAHs) were surveyed in Iranian roasted and fried chicken in Bushehr restaurants. For data collection, 73 samples (including 36 Roasted chicken and 37 Fried chicken samples) were collected from local restaurants and various retail outlets of Bushehr, Iran. The concentrations of 16 PAHs were determined by gas chromatography-mass spectrometry (GC–MS). Results indicated that the total PAHs level was ranged from 4.20 to 32.29 mg/kg and 2.06–19.65 µg/kg in Roasted and Fried chicken, respectively. The differences in PAHs levels were observed among charcoal and gas-roasted samples as well as between chicken fried with different oil types. benzo[a] pyrene(BaP), an appropriate marker for occurrence and potential toxicity of PAHs food matrixes was found in all roasted samples ranged from 1.41–5.71 mg/kg and all fried samples in range of 0.9–3.32 µg/kg. Moreover, unsaturated fatty acid (UFA) and saturated fatty acid (SFA) ratios in the in the used vegetable oils had an important role in the generation of PAHs in fried chicken.

**Specifications table**

**Table:** 

Subject area	Food hygiene
More specific subject area	Food chemistry
Type of data	Tables and figures
How data was acquired	In this data article, 73 samples (including 36 Roasted chicken and 37 Fried chicken samples) were collected from local restaurants and various retail outlets of Bushehr, Iran. The concentrations of 16 PAHs were determined by gas chromatography-mass spectrometry (GC–MS).
Data format	Raw, analyzed
Experimental factors	The analysis of all the 73 purchased samples was carried out by duplicate for PAHs.
Experimental features	The sampling method, samples transfer to the laboratory, samples preparation and analysis of them were performed according to standard method that provided in valid references.
Data source location	Bushehr city, Iran
Data accessibility	Data are included in this article
Related research article	G.Perelló, R.Martí-Cid, V.Castell, J.M. Llobet, J.L.Domingo, Concentrations of polybrominated diphenyl ethers, hexachlorobenzene and polycyclic aromatic hydrocarbons in various foodstuffs before and after cooking, Food Chem. Toxicol. 47(2009)709-15 [1].

**Value of the data**

• Up to now, there is no published work in Iran to determine the concentration of PAHs in mentioned chicken cooked by domestic restaurants. This is the first research carried out to assess the levels of PAHs in Iranian Roasted and Fried chicken.
• The obtained data of present data article can be a basis for the development of similar future studies.
• The data of this data article showed that common roasting (either gas or charcoal fuel) and frying method (using either SO or PO oils) in Bushehr restaurants could generate PAHs in chickens food.
• The acquired data showed that Benzo[a] pyrene, an indicator for occurrence of PAHs in foods, was found in all chicken samples with the lowest and highest levels detected in gas roasted chicken and charcoal roasted chicken, respectively.

## 1 Data

### 1.1 PAHs in roasted chickens

**Fig. 1:**
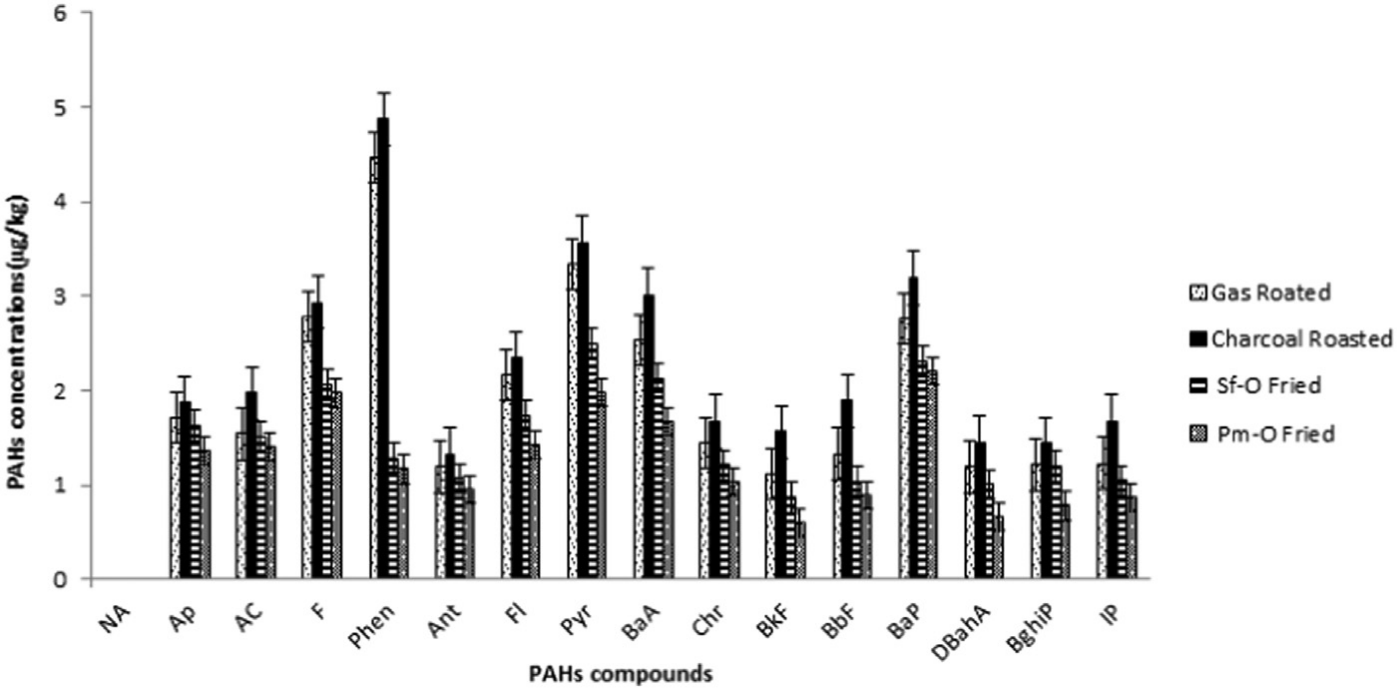
The mean concentration of PAHs compounds in roasted and fried chicken samples.

The average and range of concentration in PAHs (mg kg^−1^), and the relative percentage of each PAHs to ƩPAHs in 73 samples (including 36 Roasted chicken and 37 Fried chicken samples) analyzed are given in Fig. 1 and Table 2. As shown in these table and figure, the PAHs were categorized into low molecular weight PAHs (L-PAHs) including Ap, Ac, F, Phen and Ant and high molecular weight PAHs (H-PAHs) including Fl, BaA, Chr, Pyr, BbF, BkF, BaP, IP, DBahA, and BghiP. The levels of H-PAHs in the chicken samples roasted on charcoal were in range of 10.23 to 32.29 µg/kg, whereas this range in the chicken samples prepared by gas roasting was 9.27–25.93 µg/kg. Regarding to concentrations of L-PAHs, there was not significantly different among charcoal and gas roasted samples. The most predominant compounds between the PAHs were observed to be phenanthrene, in range of 1.45–6.21 mg/kg.

The findings in Table 2 show that BaP was found in all charcoal and gas roasted samples ranged from 1.41–5.71 mg/ kg. The lowest level of BaP (1.41 mg kg1) was observed in one of the gas roasted chicken, and the highest level (5.71 µg /kg) was found in one of the charcoal roasted chicken.

### 1.2 PAHs in Fried chickens

The addition of oil in cooking procedure causes an increase in the PAHs concentrations in chicken samples due to fat being the main precursor of PAHs [2]. In this study, we evaluated this parameter and determined values of PAHs in fried chicken samples, which are cooked using two type of oil (sunflower (SO) and palm (PO)) are presented in Table 2. As shown in Table 2, the concentrations of H-PAHs in the chicken samples fried on SO were ranged from of 5.36- 19.65 µg/kg, whereas this range in the chicken samples prepared by PO frying was 4.76–17.65 µg/kg. Regarding to concentrations of L-PAHs, the levels of L-PAHs in SO fried samples were ranged from of 2.28–11.65 µg /kg, whereas this range in the chicken samples fried by PO was 2.06–11.21 µg/kg. As can be seen, the concentrations of both H-PAHs and L-PAHs in the chicken samples fried on SO are much higher than PO fried samples.

## 2 Experimental design, materials and methods

### 2.1 Standards and reagents

All solvents (including acetonitrile, dichloromethane, methanol and n-hexane) employed in this research were high-performance liquid chromatography (HPLC) grade and obtained from Merck (Darmstadt, Germany). A mixture standard of PAHs containing 16 compounds (Table 1) were prepared by diluting100 µg of each PAH in 1 ml of solvent was and stored in −4 °C. citric acid, sodium sulfate anhydrous, potassium hydroxide and potassium persulfate came from Sigma-Aldrich (MO, USA). All glassware was washed in liquinox soap solution and then rinsed with distilled water. In order to avoiding from airborne pollution, glassware was covered with heat-cleaned aluminum foil [3], [4], [5], [6], [7].

**Table 1:** List of PAHs reported, abbreviations, formula, molecular weights, vapor pressure.

**PAH compounds**	**Abbreviation**	**Formula**	**Molecular weight**	**Vapor pressure (mm Hg)a**
**2-Rings, C10**				
Naphthalene	NA	C10H8	128	8.5 × 10^−2^
**3-Rings, C12-C14**				
Acenaphthylene	Ap	C12H10	154	2.2 × 10^−3^
Acenaphthene	Ac	C12H10	154	2.2 × 10^−3^
Fluorine	F	C12H10	37	5.3 × 10^−3^
Phenanthrene	Phen	C14H10	178	6.8 × 10^−4^
Anthracene	Ant	C14H10	178	1.7 × 10^−5^
**4-Rings, C16-C18**				
Fluoranthene	Fl	C16H10	202	5 × 10^−6^
Benzo[a]anthracene	BaA	C18H12	228	2.2 × 10^−8^
Chrysene	Chr	C18H12	228	6.3 × 10^−7^
**5-Rings, C20**				
Pyrene	Pyr	C20H12	252	5.7 × 10^−9^
Benzo[b]fluoranthene	BbF	C20H12	252	5 × 10^−7^
Benzo[k]fluoranthene	BkF	C20H12	252	5.59 × 10^–11^
Benzo[a]pyrene	BaP	C20H12	252	5.6 × 10^−9^
**6-rings, C22**				
Dibenz[a,h]anthracene	DBahA	C22H14	278	1.0 × 10^−10^
Benzo[ghi]perylene	BghiP	C22H12	276	1.03 × 10^−10^
Indeno[1,2,3-cd]pyrene	Ip	C22H12	276	10^–11^–10^−6^

**Table 2:** Determined values (Mean ± SD) for total PAHs, L-PAH, H-PAH and BaP concentrations (µg /kg) in roasted and fried chicken samples.

**Sample type**		**Total PAHs**	**H-PAH**	**L-PAH**	**BaP**
**Roasted chicken**	Charcoal roasted	34.75 ± 1.09	18.31 ± 0.72	12.97 ± 0.52	3.19 ± 0.41
	Gas roasted	30.01 ± 1.04	21.78 ± 0.76	11.7 ± 0.61	2.76 ± 0.37
**Fried chicken**	Palm-Oil fried	19.01 ± 0.61	15.04 ± 0.55	9.29 ± 0.34	2.31 ± 0.23
	Sun flower-oil fried	22.6 ± 0 ± 0.56	12.14 ± 0.63	6.87 ± 0.44	2.21 ± 0.31

### 2.2 Sampling and sample preparation

73 samples (including 36 Roasted chicken and 37 Fried chicken samples) were obtained from local restaurants and various retail outlets of Bushehr, Iran. The locations of retail outlets and restaurants were chosen from different areas and the sampling was done on different days. The sampling was carried out on December, 2016. Samples were collected from local restaurants, which used two type of oil (sunflower (SO) and palm (PO)) for cooking of Fried chicken and two type of fuel (gas and charcoal) for Roasted chicken. All of the purchased samples were put in special bag beside the ice and transferred to the laboratory. All samples were cut into 3–6 Cm portion, homogenized and packed in foil bags and stored at −80 °C in the dark, until use for PAHs analysis [8], [9]. Typically, all of the samples were prepared in the same day of obtain.

### 2.3 Extraction and analysis

Firstly 4 g of each sample were pulverized to fine particles and then, Iml internal standard (containing biphenyl in methanol) was added and the obtained solution was homogenized for 20 min. Then 8 mL methanol /acetonitrile (35%:v/v) and 8 mL potassium hydroxide (1 M) were added to the mixture. After this step, mixture was sonicated for 10 min at 42 °C and thereafter centrifuged at 90,000 RPM for 8 min and the fat was eliminated by filtration. Then pH of final mixture was adjusted on 6.5 using hydrochloric acid. After this step, 12 mg magnetic multiwall carbon nanotubes (MWCNTs) as an adsorbent and 490 mg NaCl were added into the each sample and then were vortex-mixed for 6 min. After that, an external magnet was used to collect the magnetic adsorbent to the side of the sample-containing tube. Supernatant was removed from the vial and then 6 mL dichloromethane was added to extract the PAHs from the adsorbent with vortex-mixing for 4 min. Then, magnetic adsorbent was gathered to the side of the sample-containing tube repeatedly and the solvent was re-concentrated and evaporated under a smooth flow of nitrogen gas. The dried residue was dissolved in 50 µL of methanol-acetonitrile (50:50 v/v) and the mixture was shaken via vortex-mixer for 2 min [10], [11], [12], [13], [14]. Finally, 1 mL of the resulted solution was taken and injected into the gas chromatography-mass spectrometry (GC–MS) (Agilent 7890A- 5975C, USA), equipped with a DB-5MS capillary column. Carrier gas in this procedure was helium (purity > 99.999%) at a constant flow rate of 1 ml/min. The analysis of all the 73 purchased samples was carried out by duplicate for PAHs.

### 2.4 Statistical analyses

All of the runs were carried done, at least, in duplicate and the average of values selected for statistical interpretation. Before analyzing, the normality of all data was checked with the Shapiro–Wilk Test. SPSS for windows version 22 (SPSS Inc, Chicago, USA) was used to ANOVA and Duncan test. Statistical significance was set at *p* < 0.05. The findings data were presented as average values ± standard deviations from replicates.

## References

[1] Perelló G., Martí-Cid R., Castell V., Llobet J.M., Domingo J.L. (2009). Concentrations of polybrominated diphenyl ethers, hexachlorobenzene and polycyclic aromatic hydrocarbons in various foodstuffs before and after cooking. Food Chem. Toxicol..

[2] Farhadian A., Jinap S., Abas F., Sakar Z.I. (2010). Determination of polycyclic aromatic hydrocarbons in grilled meat. Food Control..

[3] Pirsaheb M., Fattahi N. (2015). Trace determination of heavy metals in farmed trout fish using dispersive liquid–liquid microextraction based on solidification of floating organic drop and graphite furnace atomic absorption spectrometry. Anal. Methods..

[4] Safari Y., Karimaei M., Sharafi K., Arfaeinia H., Moradi M., Fattahi N. (2018). Persistent sample circulation microextraction combined with graphite furnace atomic absorption spectroscopy for trace determination of heavy metals in fish species marketed in Kermanshah, Iran, and human health risk assessment. J. Sci. Food Agric..

[5] Shamsipur M., Fattahi N., Pirsaheb M., Sharafi K. (2012). Simultaneous preconcentration and determination of 2, 4‐D, alachlor and atrazine in aqueous samples using dispersive liquid–liquid microextraction followed by high‐performance liquid chromatography ultraviolet detection. J. Sep. Sci..

[6] Sadeghi M., Nematifar Z., Fattahi N., Pirsaheb M., Shamsipur M. (2016). Determination of bisphenol A in food and environmental samples using combined solid-phase extraction–dispersive liquid–liquid microextraction with solidification of floating organic drop followed by HPLC. Anal. Methods..

[7] Pirsaheb M., Fattahi N., Shamsipur M., Khodadadi T. (2013). Application of dispersive liquid–liquid microextraction based on solidification of floating organic drop for simultaneous determination of alachlor and atrazine in aqueous samples. J. Sep. Sci..

[8] Chung S.Y., Yettella R.R., Kim J.S., Kwon K., Kim M.C., Min D.B. (2011). Effects of grilling and roasting on the levels of polycyclic aromatic hydrocarbons in beef and pork. Food Chem..

[9] Raeisi A., Arfaeinia H., Seifi M., Shirzad-Siboni M., Keshtkar M., Dobaradaran S. (2016). Polycyclic aromatic hydrocarbons (PAHs) in coastal sediments from urban and industrial areas of Asaluyeh Harbor, Iran: distribution, potential source and ecological risk assessment.

[10] Alomirah H., Al-Zenki S., Al-Hooti S., Zaghloul S., Sawaya W., Ahmed N., Kannan K. (2011). Concentrations and dietary exposure to polycyclic aromatic hydrocarbons (PAHs) from grilled and smoked foods. Food control.

[11] Arfaeinia H., Kermani M., Hashemi S.E. (2017). Concentrations and potential risk assessment of polycyclic aromatic hydrocarbons (PAHs) from indoor dust of Bushehr, Iran. Glob. Nest J..

[12] Akpambang V.O.E., Purcaro G., Lajide L., Amoo I.A., Conte L.S., Moret S. (2009). Determination of polycyclic aromatic hydrocarbons (PAHs) in commonly consumed Nigerian smoked/grilled fish and meat. Food Addit. Contam..

[13] Yousefi M., Shemshadi G., Khorshidian N., Ghasemzadeh-Mohammadi V., Fakhri Y., Hosseini H., Khaneghah A.M. (2018). Polycyclic aromatic hydrocarbons (PAHs) content of edible vegetable oils in Iran: a risk assessment study. Food Chem. Toxicol..

[14] Heshmati A., Ghadimi S., Khaneghah A.M., Barba F.J., Lorenzo J.M., Nazemi F., Fakhri Y. (2018). Risk assessment of benzene in food samples of Iran׳s market. Food Chem. Toxicol..

